# Giant abdominal desmoid-type fibromatosis

**DOI:** 10.4322/acr.2024.471

**Published:** 2024-02-01

**Authors:** Saikat Mitra, Amitava Dutta

**Affiliations:** 1 Unipath Speciality Laboratory Limited, Department of Histopathology and Cytopathology, Kolkata, West Bengal, India

**Keywords:** Fibromatosis, Aggressive, Leiomyoma, Abdominal Neoplasms, Cesarean Section, Myofibroblasts

Desmoid-type fibromatosis (DF) is a locally aggressive myofibroblastic tumor accounting for approximately 0.03% of all neoplasms and less than 3% of all soft tissue tumors.^1^ The estimated incidence in the general population is 2 to 4 cases per million per year. In the past, this lesion was considered as an aberrant healing response against local trauma, irritation, or inflammation; however, later a clonal proliferation of the spindle cell was confirmed, and local recurrence as well as distant metastasis was identified.^2^ DF affects 10%-20% of patients with familial adenomatous polyposis. Sporadic DF predominantly affects young adults, especially women, and it is often related to pregnancy.^3^ Although it may occur in nearly every body part, it usually involves the extremities and trunk, including the abdominal wall and intra-abdomen. Intra-abdominal desmoid tumors are mainly located within the mesentery or pelvis.^4^ A bulky desmoid tumor located in the pelvis or lower abdominal wall near the uterus may be preoperatively suspected as a large subserosal uterine leiomyoma.

Genetic alterations in the*APC*and*CTNNB1*genes are thought to be vital events resulting in hereditary and sporadic DF. Sporadic DF is associated with a high incidence (85%) of mutations in the*CTNNB1*gene, which may lead to uninhibited activation of the Wnt pathway and the accumulation of excessive cytoplasmic β-catenin, ultimately resulting in tumor development.^5^

The early diagnosis of DF is challenging. The findings of computed tomography (CT) and magnetic resonance imaging (MRI) can guide the decisions regarding patient management, but the ultrasound features of DF are non-specific. The relationship between the tumor and the adjacent structures should be carefully evaluated to decide the feasibility of surgery.^6^

Complete surgical resection is usually the standard treatment in patients with resectable desmoid tumors. However, the local recurrence rate is still high after resection alone due to its infiltrative pattern and positive margins after surgical resection.^7^

Radiotherapy is another treatment modality for desmoid tumors, but its efficiency is controversial. Recently published meta-analysis and the review suggested the benefit of postoperative radiotherapy over surgery alone in long-term cure and prevention of recurrence.^8^ Low-dose chemotherapy or systemic therapy (such as hormonal therapy, non-steroidal anti-inflammatory therapy, or targeted therapy) in patients with advanced unresectable tumors or recurrent disease after surgery and/ or radiotherapy is also used.^9^

A 24-year-old lady presented with gradually progressive pain in the whole abdomen for six months. She had a prior history of lower uterine cesarean section done 3 years back and a history of missed abortion 4 years back. On per-abdominal examination, a 10x8 cm large mass was palpated mainly in the right lower abdomen, with side-to-side mobility. The speculum examination showed a smooth cervix. Per-vaginal examination revealed a large mass felt through the right fornix. Abdominal computed tomography showed bilateral normal ovaries and a large heterogeneous mass measuring 11x9 cm close to the uterus. A possibility of pedunculated subserosal leiomyoma was considered. Given progressive clinical symptoms, laparotomy was performed. On per-operative examination, a large mass was identified as closely adhered to the mesentery of the right colon. The uterus and bilateral adnexa were free of tumor. The tumor was excised in toto with the right colon.

**Figure 1:**
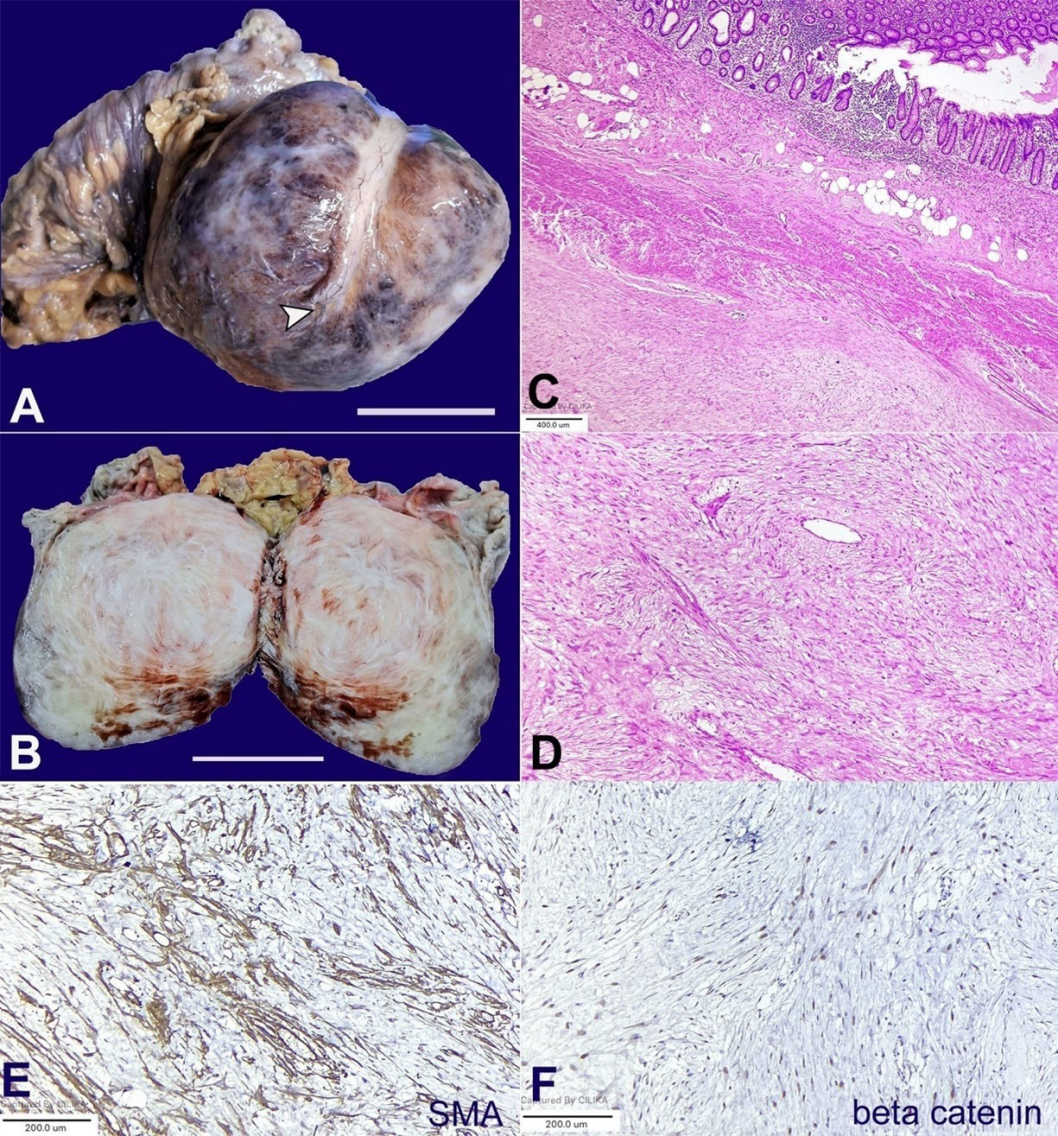
**A** - Gross view of the giant globular tumor with a smooth surface and closely adhered to the right colon and appendix;the white arrowhead points towards the tip of the adhered appendix (scale bar= 5 cm); **B** - Cut surface of the tumor shows a solid, well-circumscribed whitish tumor with areas of trabeculations (scale bar= 5 cm); **C** - Photomicrograph shows a low-grade spindle cell mesenchymal tumor of low cellularity densely adhered to the serosal surface of the appendix with infiltration of muscularis of the appendix (H&E,40x); **D** - Photomicrograph shows spindle cell tumor with bland elongated nuclei and moderate cytoplasm disposed over a hyalinized and myxoid stroma interspersed with thin-walled vessels. (H&E, 100x); **E** - Photomicrograph shows tumor cells positive for smooth muscle actin (SMA) immunohistochemistry (100x); **F** - Photomicrograph shows tumor cells with nuclear localization of beta-catenin (100x).

Gross examination revealed a rounded mass of size 11x9 cm with a smooth outer surface. The tumor was densely adhered to the cecum and appendix and had engulfed the appendix (Figure 1A). The cut surface shows a solid, whitish, firm lesion with prominent trabeculations (Figure 1B).

Microscopic examination revealed a mesenchymal tumor with low cellularity, composed of bland spindled cells over a densely collagenized matrix interspersed with thin vascular channels (Figure 1D). Mitosis, necrosis, and significant atypia were absent. The tumor was adhered within the serosal surface of the appendix and colon and also showed infiltration of the retroperitoneal skeletal muscle fibers (Figure 1C). On IHC, the tumor cells were positive for Vimentin, Smooth muscle actin (SMA) and showed nuclear localization of beta-catenin with cyclinD1 (Figures 1E and 1F). IHC for h-caldesmon, S-100, CD117, DOG-1, myogenin, ALK, and STAT-6 were negative, ruling out other differential diagnoses, including leiomyoma, nerve sheath tumor, gastrointestinal stromal tumor, spindle cell rhabdomyosarcoma, inflammatory myofibroblastic tumor, and solitary fibrous tumor. Ki-67 labeling was ~2-3%. The DF diagnosis was rendered based on the histology and IHC features.

Our patient was advised postoperative close follow-up with clinical and imaging modalities at regular intervals.

In summary, awareness about this rare tumor in intra-abdominal locations in young female patients with a prior history of childbirth and surgery and its possible clinical and imaging overlap with leiomyoma prevents aggressive treatment in DF patients. A pre-operative biopsy can confirm the diagnosis. However, in symptomatic patients, surgery is the mainstay of therapy. A search for possible CTNNB1 and APC gene mutation is vital.
